# Ethical Considerations for Wildlife Reintroductions and Rewilding

**DOI:** 10.3389/fvets.2020.00163

**Published:** 2020-04-03

**Authors:** Carl-Gustaf Thulin, Helena Röcklinsberg

**Affiliations:** ^1^Department of Anatomy, Physiology and Biochemistry, Swedish University of Agricultural Sciences, Uppsala, Sweden; ^2^Department of Animal Environment and Health, Swedish University of Agricultural Sciences, Uppsala, Sweden

**Keywords:** restoration, conservation, reintroduction, rewilding, ecosystem service, ethics, animal welfare

## Abstract

The recovery of many populations of large carnivores and herbivores in major parts of Europe and North America offers ecosystem services and opportunities for sustainable utilization of wildlife. Examples of services are hunting, meat, and skin, along with less invasive utilization such as ecotourism and wildlife spotting. An increasing number of studies also point out the ecosystem function, landscape engineering, and cascading effects of wildlife as values for human existence, biodiversity conservation, and ecosystem resilience. Within this framework, the concept of rewilding has emerged as a means to add to the wilderness through either supplementary release of wildlife species already present or reintroduction of species formerly present in a certain area. The latter involves translocation of species from other geographical areas, releases from captivity, feralization, retro-breeding, or de-domestication of breeds for which the wild ancestor is extinct. While all these initiatives aim to reverse some of the negative human impacts on life on earth, some pose challenges such as conflicts of interest between humans and wildlife in, for example, forestry, agriculture, traffic, or disease dynamics (e.g., zoonosis). There are also welfare aspects when managing wildlife populations with the purpose to serve humans or act as tools in landscape engineering. These welfare aspects are particularly apparent when it comes to releases of animals handled by humans, either from captivity or translocated from other geographical areas. An ethical values clash is that translocation can involve suffering of the actual individual, while also contributing to reintroduction of species and reestablishment of ecological functions. This paper describes wildlife recovery in Europe and North America and elaborates on ethical considerations raised by the use of wildlife for different purposes, in order to find ways forward that are acceptable to both the animals and humans involved. The reintroduction ethics aspects raised are finally formulated in 10 guidelines suggested for management efforts aimed at translocating wildlife or reestablishing wilderness areas.

## Introduction

Human domination on earth has influenced the conditions for life and the long-term existence of all living organisms for thousands of years (1). This has resulted, e.g., in a 58% decline in population abundance of 3,706 species monitored between 1970 and 2012 (2). Large mammalian carnivores and herbivores have been notably negatively affected by human activities (3–7). The consequences of these declines are a trophic downgrading of the planet (8–10). However, there are exceptions to these negative scenarios for large mammals in many areas of Europe and North America. After a long period of decline, which started with the first appearance of our species, *Homo sapiens*, outside Africa (11), mostly as an effect of human hunting (12–14), there has been a dramatic recovery in wildlife in Europe and North America during the past 50 years [e.g., (15–18)]. This recovery is largely a result of protection from hunting, limitations on toxic waste release, changes in land management, and an increase in protected areas/reserves. Translocation of species, as introductions, reintroductions, or supplementary releases, has also contributed.

**Figure 1:**
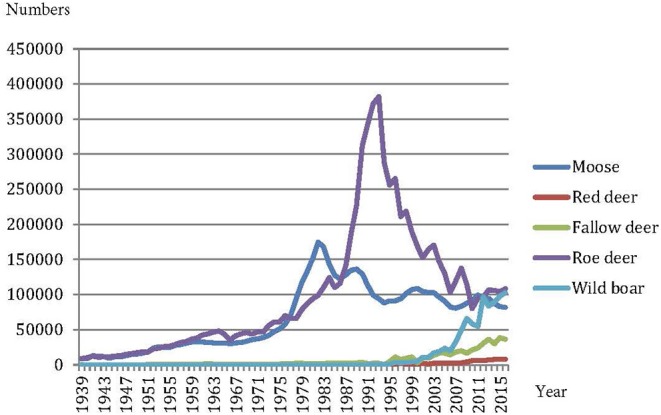
Game bags of ungulates in Sweden from 1939–2016 (The Swedish Association for Hunting and Wildlife Management—Wildlife Monitoring, www.vildata.se; 2018-07-27).

In Sweden, ungulate populations decreased to a minimum in the mid-nineteenth century since the Swedish king Gustav III decided in 1789 to open hunting rights to all landowners (19); fewer than 100 individuals for red deer (*Cervus elaphus*) and roe deer (*Capreolus capreolus*), and probably fewer than 1,000 for moose (Table 1). Wild boar (*Sus scrofa*) became extinct. Fallow deer (*Dama dama*) may have occurred sporadically on some larger estates in the south of Sweden. Large carnivore numbers also plummeted during the early twentieth century (18). In addition, the Eurasian beaver (*Castor fiber*) became extinct (28) and the European otter (*Lutra lutra*) population came under pressure (29). In 1830, the formation of Swedish Association for Hunting and Wildlife Management (Sw. “*Svenska Jägareförbundet*”) was a turning point for the overall conservation of populations of large mammals and birds in Sweden. However, it took almost 100 years before the populations of large ungulates started to make a substantial comeback in Sweden. A combination of careful management and selective hunting, whereby primarily juveniles and younger specimens were shot, and implementation of novel forest management routines resulted in large amounts of suitable forage in the landscape (30) and facilitated the recovery of, first, the moose population, which peaked in the 1980's, and, second, the roe deer population, which peaked in the 1990's. This population development is recognizable in the Swedish game bag statistics, compiled by the Swedish Association for Hunting and Wildlife Management (Figure 1). The wild boar was reintroduced [cf. (24)], and populations are still growing, along with red deer populations aided by supplementary release of non-native contingents (cf. (31)). Fallow deer are still expanding in both range and numbers, and during the past 10–20 years, populations of mouflon (wild sheep; *Ovis orientalis*) have started to appear in many places (P. Kjellander, 2007; unpublished results). Another large herbivore comeback worth mentioning is the Eurasian beaver, which numbers around 100,000 today thanks to a successful reintroduction effort that started in 1922 (25).

**Table 1:** Approximate minimum and current estimates of population numbers of a selection of wildlife species in Sweden.

**Species**	**Year**	**Number**	**References**
Moose (*Alces alces*)	1840	Few (no estimates available)	(20)
	2016	240,000	(20)
Red deer (*Cervus elaphus*)	1840	<100	(21)
	2016	26,000	(22)
Roe deer (*Capreolus capreolus*)	1840	<100	(23)
	2016	300,000	(20)
Wild boar (*Sus scrofa*)	1976	0	(24)
	2018	350,000	H. Thurfjell, pers. comm.
European beaver (*Castor fiber*)	1922	0	(25)
	1995	>100,000	(26)
Wolf (*Canis lupus*)	1970	0	Swedish EPA
	2018	305	Swedish EPA
Brown bear (*Ursus arctos*)	1930	130	Swedish EPA
	2013	2,800	Swedish EPA
Lynx (*Lynx lynx*)	1920	Few (no estimates available)	Swedish EPA
	2018	1,200	Swedish EPA
Wolverine (*Gulo gulo*)	1960	100	Swedish EPA
	2017	522	Swedish EPA

*In addition to the large ungulates included below, 38,860 fallow deer (Dama dama) are shot on annual basis (Figure 1), indicating a population of 126,000 individuals (22), and mouflon (Ovis orientalis) occur locally in south and central Sweden, likely numbered in thousands (cf. P Kjellander, 2007, unpublished data). There is also a local population of 11 wild muskoxen (Ovibos moschatus) in the south part of the Swedish mountains (B. Warensjö, pers. comm.), and sporadically white-tailed deer occur at the northern border to Finland [cf. (27)]*.

An important factor influencing the population growth of large ungulates and herbivores during the first half of the twentieth century was the absence of large carnivores. When ungulates (i.e., wild prey) became rare in the nineteenth century, large-scale carnivore predation on livestock became an increasing problem. This led to bounty hunts and organized population control of large carnivores, often with the intention to exterminate (20). The bounty for wolf (*Canis lupus*) ended finally in 1965, and since January 1966, this species has been fully protected (32). By 1965, the wolf had nearly disappeared and was declared extinct in Sweden in 1980 (32). Brown bear (*Ursus arctos*), Eurasian lynx (*Lynx lynx*), and wolverine (*Gulo gulo*) populations also fell to a minimum at that time (18). The protection of large carnivores was introduced primarily following actions from conservation organizations such as the Swedish Society for Nature Conservation (Sw. “*Naturskyddsföreningen*”), which was formed in 1909. By the time large carnivore protection was implemented, the populations of large ungulates to prey upon had recovered, providing an essential foundation for large carnivore population recovery. Thus, although large carnivore protection was not a major concern for hunters in Sweden, the synergetic effects of actions by hunters and conservationists benefited overall wildlife recovery. The recovery of forest-dwelling mammals in Sweden is documented in the Swedish Red List report (33), an interesting contrast to the rapid extinction rate globally (34).

The wildlife recovery in Sweden may be exceptional, which is why we use the Swedish example as a reference scenario in this paper. However, as indicated above, there are reports of similar recovery patterns in other parts of Europe [cf. (16, 35, 36)]. Despite ongoing negative trends for some species or groups of species in Europe, the overall trend in the Palearctic is a 6% increase in monitored vertebrates since 1970 (16). The “*Wildlife Comeback in Europe*” report by Deinet et al. 14 records positive population trends for a number of European species. Several of Europe's large carnivores are increasing in numbers over wide ranges [e.g., (18, 37, 38)]. Similarly, large herbivores are increasing in numbers, partly because of protection and regulated hunting, but also through active restoration initiatives and reintroductions [e.g., (39–41)]. Species such as European bison (*Bison bonasus*), moose, red deer, wild boar, European brown bear, gray wolf, Eurasian lynx, and Eurasian beaver are all showing positive population trajectories, and have done so for the past 50 years. The explanatory factors for these recent comebacks are of course different conservation actions and initiatives, but many of these large mammals have also benefited from ongoing land abandonment and urbanization in less populated parts of Europe, in particular Eastern Europe (42).

Similarly, in North America, many species of large carnivores and herbivores have made a recent comeback, strengthening in population size, distribution, and conservation status (17). The recovery of North American wildlife is often attributed to the 26th president of the United States, Theodore Roosevelt. He institutionalized and popularized conservation, and expanded federal protected lands by creating the United States Forest Service (43), an initiative that still permeates North American conservation and wildlife management (44). The stipulation that wildlife is a public trust and concern, and that hunting is an obligation governed by legitimate purposes, rather than a market-induced activity, has been referred to as the North American model of wildlife conservation (45). Species such as black-tailed deer (*Odocoileus hemionus* ssp. *columbianus*), white-tailed deer (*O. virginianus*), mule deer (*O. hemionus*), and black bear (*Ursus americanus*), which were on the brink of extinction in the late nineteenth century, now occur in vast areas of the United States and Canada; American bison (*Bison bison*) again roam large areas of the great plains; and moose, red deer, and pronghorn (*Antilocapra americana*) are widely occurring, as are large carnivores such as brown bear, gray wolf, and mountain lion (*Puma concolor*).

In this paper, we take the above-described situation as a point of departure for an ethical reflection of aspects relevant in reintroductions, translocation, and overall wildlife management. Our focus lies on considerations evoked by the current situation regarding ecosystem services, rewilding, and wildlife dependence each presented in a separate section. Further, we take an explicit ethical view on what is needed to ensure that wildlife management honors ethical standards and handles challenges inherent to translocations and introductions in a professional way. We suggest 10 ethical guidelines for management efforts aimed at translocating wildlife or reestablishing wilderness areas.

## Ecosystem Services

The concept of “environmental services” was first introduced by Wilson and Matthews (46), renamed “ecosystem services” by Ehrlich and Mooney (47), and gained broader attention after the signing of the Convention on Biodiversity (CBD) in 1992 (48). The CBD refers to a set of measures to aid biodiversity conservation by emphasizing the intrinsic and actual value and importance of natural resources and ecosystem functions. The economic value of the world's collective ecosystem services was estimated at 125–145 trillion USD/year by Constanza et al. (49).

The classical subdivision of ecosystem services is into provisioning, regulating, cultural, and supporting services (50). Provisioning services are generally described as what humans need to subsist, like food, fresh water, wood, and fiber. The regulating services are wider, relating to impacts on climate, water systems, and disease dynamics, while the cultural services are naturally anthropogenic in their context, for example, esthetic, spiritual, educational, or recreational. The supporting services are fundamental ecosystem functions such as primary production, nutrient cycling, and soil formation. Many of the ecosystem services also relate to the 17 Sustainable Development Goals (SDGs) defined by United Nations (51), in particular SDG 13 (Climate action) SDG 14 (Life below water), and SDG 15 (Life on land), but also SDG 1 (No poverty), SDG 2 (Zero hunger), SDG 3 (Good health and well-being), and SDG 6 (Clean water and sanitation). Thus, the ecosystem services provided by large, wild animals are a critical concern for humanity at large.

Wildlife offers opportunities for provisioning ecosystem services such as meat, skin and fur, down and feathers, antlers and trophies, along with less invasive services such as ecotourism and wildlife spotting (52–54). Although provisioning services may be an underlying objective in wildlife management, recovered populations of large carnivores and herbivores in parts of Europe and North America also provide regulating services such as predation or grazing. Both these are important aspects of biodiversity conservation and ecosystem function, as recognized in a recent restoration strategy called rewilding (see below). In addition, or in combination, wildlife has an impact on supporting services such as nutrient, carbon, and water dynamics, and generally facilitates ecosystem resilience (55, 56). Wildlife may also function as climate change mitigators (55, 57–59). The cultural services that wildlife provide may be the most important, since presence of wildlife in a landscape adds to a notion of biological richness that provides comfort and beauty, but also a sense of food security and resource stability, as well as evoking interest in biodiversity and sustainable landscape management.

## Rewilding

The concept of rewilding was first formulated by Soulé and Noss (60) and Barlow (61) as a positive trajectory for conservation and evolution that, in addition to protection of species, also included restoration of the degenerated ecosystem of other non-marginal species. It emerged from the gradual realization that humans throughout time, i.e., not only in recent centuries or millennia, but over tens of thousands of years, have depressed and exterminated many large species of birds and mammals [e.g., (11, 62, 63)]. Rewilding aims to enhance wilderness through supplementary release of wildlife species already present and through reintroduction of species formerly present. The latter can be achieved by translocation of species from other geographical areas, actual feralization (rewilding), or retro-breeding (into ancestral phenotypes or genotypes) of domestic strains for which the wild ancestor is extinct, such as cattle and horses. Recent developments in genetics and animal breeding offer biotechnical opportunities to retro-breed extinct species [e.g., (64–67)].

An important aspect of the rewilding initiative is to enable tourism that benefits both local inhabitants and visitors, i.e., sustainable ecotourism (35, 36). Ecotourism has potential as an economic revenue and may provide incitement for conservation, but there may be concerns for restrictions associated with hunting, agriculture, and forestry. Rewilding that aims for ecotourism may however be combined with hunting opportunities [cf. (52)]. According to recent figures from the Swedish Board of Agriculture, Swedish consumption of total (both domestic and wild) terrestrial meat (i.e., no aquatic animals) is 85.5 kg/person/year, of which wildlife meat comprises around 2 kg/person/year on average (68). Thus, wildlife meat is a significant amount of the overall meat consumption in Sweden. A reduction in per-capita meat consumption, accompanied by an increase in number of wildlife and subsequent (sustainable) harvest, may enable Sweden to source an even higher proportion of the meat consumed from wildlife, an interesting opportunity given the global challenges related to conservation of species, climate change, and food production. However, a society dependent on wildlife as a food resource raises ethical questions related to “harvest” through hunting, with obvious risks of unintended harm to a larger number of hunted specimens compared with farming of animals accustomed to humans. On the other hand, if wildlife were to provide an extensive amount of the animal protein needed in a sustainable system, ecosystems would potentially be more resilient to human presence. Further, the welfare and integrity of wild animal can be regarded as less compromised by humans than the welfare of intensively reared poultry, pigs, and ruminants. Moreover, as the numbers of bred and killed animals would decrease radically, far fewer animals would be affected by potential welfare impairments.

In addition, there is increasing interest in the opportunities for restoration of trophic levels, ecosystem function, and resilience that may accompany different rewilding initiatives, typically referred to as “trophic rewilding” [e.g., (9)], as a countermeasure to the trophic degradation eloquently described by Estes et al. (8). Svenning et al. (69) define trophic rewilding as “*species introductions to restore top-down trophic interactions and associated trophic cascades to promote self-regulating biodiverse ecosystems*.” There are numerous examples of reintroductions having positive impacts on ecosystems, such as the trophic cascades attributed to the reintroduction of wolves in Yellowstone National Park (70), the ecological impact of white rhinoceros in Kruger National Park (71), and the wetland creation resulting from introduction of beaver in Europe (72, 73) and North America (74, 75). Few experimental studies have addressed the implications of trophic rewilding thus far, but a recent study of the ecological impact of horses kept under feral conditions reported inhibition of shrubification (76) and benefits for plant and insect diversity (77).

The processes and initiatives that accompany rewilding attempts can generally be regarded as positive, in that they aim to restore the negative impact that human domination has imposed on life on earth, and is still imposing in many places. However, a rewilding process can also pose challenges, such as conflicts of interest between humans and wildlife in forestry, agriculture, traffic, or disease dynamics (e.g., zoonosis). These challenges are mainly economic (e.g., damage to forestry, agriculture, and horticulture), while others relate to human health and welfare (e.g., traffic incidents, disease dynamics). An additional consequence of the utilization of wildlife as a resource, irrespective of the specific form (e.g., hunting, ecotourism, ecosystem function), is how wildlife itself reacts to the rewilding process. Inevitably, some individuals will suffer and die during reintroduction efforts, but the extent may differ between methods, which justifies a proactive risk assessment.

## Wildlife Dependence

Human domination on earth, the impact of which extends to all aspects of other living organisms, places humans in a responsible position as regards utilization of the ecosystem services provided by, e.g., wildlife, such as hunting, meat, skin, ecotourism, and wildlife spotting (78). Since humans are able to exterminate, preserve, or support most other life forms, and are capable of reflecting (and socially expected to reflect) upon their actions, they have particular responsibility for life on earth [e.g., (79–81)]. This logic underlies much of the CBD and conservation actions overall, and thus provides an ethical framework for conservation, and also for utilization of wildlife services.

The rewilding approach raises interesting, important, and, perhaps surprisingly, ethical and legal issues not foreseen or previously perceived within wildlife management. Under current legislation in many countries, humans are responsible for the welfare of domestic animals in their care (82, 83). This means that domestic animals, most of which are housed or fenced in, have the right to food and water, shelter from the weather, protection from predation, and also disease protection and treatment. In line with the legislation for domestic animals, but from an ethical point of view, a series of marginal situations arise in conventional wildlife management, for example, when humans protect wildlife from predation, provide supplementary feeding, interfere in reproduction, provide shelter, or shape landscapes to benefit certain species. Thus, restoration that aims to be beneficial for certain species or individuals also places the restorer in a potential caretaking position, where the level of ethical responsibility for the welfare of an individual animal might be difficult to discern.

Rewilding leads to additional ethical issues, as it blurs the boundary between wild and domestic even further (84). As reintroduction and rewilding in practice means introduction, or at least supplementary release, of individuals into new environments, humans compromise the welfare of these individuals in different ways. First, rewilding of domesticated animals (such as cattle or horses) may include transportation to a certain habitat and then leaving the animals to take care of themselves. Second, during translocation of wild animals, catching, handling, and transportation can impact welfare. Third, in potential cases of retro-breeding (i.e., introducing a number of individuals purposely bred as a “re-creation” of an extinct species, such as aurochs), the individuals are handled by humans, albeit in extensive farming conditions, before being left in the wild. In all three cases (introduction/supplemental release, feralization/rewilding, and retro-breeding), the animals involved cross the boundary between being human-reared and wild. If handled as domesticated animals, albeit briefly, wild individuals with no prior experience of humans risk experiencing stronger stress than domesticated animals (85, 86). Legislation and ethics may differ in how to reflect on this, but the welfare of the individual animal may still be impaired. One possible option is to handle welfare challenges in wildlife management by the same means as suggested for wildlife research, through application of the two of the 3Rs (replacement, reduction, and refinement), i.e., except for replacement strive for reduction of number of affected animals and refinement of methods and welfare impairment (87).

Many wildlife releases inevitably lead to suffering at different levels of the released specimen that has to encounter and adapt to a novel habitat, seek forage, and seek protection from weather and predation. If unsuccessful, released specimens may even die of starvation because they are unfamiliar with the new area, are preyed upon by local predators, or are harassed by existing members of the same or a related species. Others may be shot during regular hunting, either by accident or because of the incidental purpose of the release as a form of “put-and-take” or to achieve more sustainable long-term establishment that aims to increase population size for hunting purposes. Released specimens may also become infected with diseases transmitted from conspecifics or closely related species, or may experience stressful situations imposed by intrusive human observers (88). In addition, released specimens may have negative effects on the already present fauna and flora (89).

Reversing the argument, however, many of the species that rewilding and reintroductions aim to restore or reintroduce became extinct because of humans, so reintroduction may be the least we can do to compensate for previous mistakes. In order to enhance biodiversity and offer a multitude of life forms on earth a fair chance to reestablish, some individuals may need to experience hardship; that is, the envisaged consequences justify the means. This utilitarian reasoning, however, pinpoints a clash of ethical values between the fundamental claim of not imposing suffering on a specimen and the purpose of restoration of anthropogenically induced extinctions through the means of translocation, i.e., the ethical question is if the release can be seen as an acceptable compensation for the first mistake of making a species extinct. If so, it can, on the other hand, be argued that we inflict harm on the entity “species” twice: first by causing its extinction and then by re-establishing it in a manner that causes its members to suffer. In that view, we can be seen as trespassing a moral boundary twice. In any case, handling the instable entity of a species, there will inevitably be different individuals that are affected by extinction and by restoration. This raises the related issue of whether compromising the welfare of one individual can be compensated for by handling another specimen in a better way. An analogous issue that lies at the core of veterinary research ethics is whether harming one individual in the process of treatment can be regarded as acceptable if it potentially leads to increased welfare for future individuals of the same species or breed. This relates to the scope of agent responsibility, moral relations, and the ethical actions that can reasonably be expected. Palmer (90) argues in favor of applying different kinds of moral relationships to wild and domestic animals, partly depending on external factors such as culture or context, applying a laissez-faire approach to wild animals (not harming, but also not actively supporting), and obeying a moral obligation to care for domesticated animals. However, Palmer (90) shows that, as humans negatively influence the habitats of wild animals and their possibilities to survive and reproduce, humans might well-impose the ethical responsibility to assist them if necessary for their survival. This line of reasoning applies even more strongly to rewilded animals. It relates to the classical conflict between environmental ethics and animal ethics (91, 92) that concerns the value of a wild individual's life and whether it is relevant to consider ethically. Being alive may be considered a value in itself, and hence creation of a new “wild” individual is morally acceptable, or instead its ecosystem role is what matters morally. In the former, retro-breeding and establishment of suitable conditions for large numbers of offspring from rewilded species is an important aim. In the latter, the overall effect on the habitat and ecosystem becomes more important. In both cases, the welfare of the animals in question matters to themselves and challenges ideas of human responsibility.

## Reintroduction Ethics

There are a few core challenges that need to be considered in the rewilding and reintroduction context. First, the objective of the effort and the potential suffering wild animals encounter in this process need to be related to the ultimate purpose of the measures taken. If beauty and pleasure for humans is the prime objective, the ethical considerations with respect to the individual animal may be weighted more strongly than if the prime reason for the action is species or ecosystem recovery, since pleasure and beauty are social values easily disrupted by the sight or awareness of animals in pain or with impaired welfare due to human actions. Similarly, if the objective is to generate more tangible short- or long-term provisional ecosystem services, such as meat or skin, it may be difficult to accept the suffering imposed by introductions or supplementary releases, as it would be a double instrumentalization of the animals, or clash with the view of hunting as a means to gain meat from animals having a good life until they die instantly. On the other hand, if the objectives are placed in a more holistic perspective, such as restoring, preserving, or saving a species, or generating ecosystem function or resilience, the suffering of specific specimens may be an acceptable cost, not on the individual level of course, but for one or many species or even ecosystems and biodiversity as a whole. We may consider this suffering “collateral damage,” a necessary evil for a greater good.

Second, restoration and rewilding may be done in very different ways, but methods that minimize the suffering of the translocated specimens should always be used (again, this is in line with ethical considerations on using animals in research and the so-called 3Rs, replacement, reduction, and refinement). The term “soft release” is often used, meaning giving the animals a chance to adapt to the surroundings prior to release. Nevertheless, quantity may sometimes be preferable to quality, and we may need to consider the basic biological prerequisites of each and every species targeted for translocation before implementation in a plausibility analysis; e.g., the suffering of thousands may be argued to be still worthwhile if the benefits are for millions. Similarly, the suffering of individuals of one species may benefit an ecosystem with a magnitude of species. Here, however, the level of suffering is also relevant, as it will differ due to habitat, translocation method chosen, animal species, and individuals.

A plausibility analysis should also include a description of the consequences of both action and passivity. It could always be argued from an animal right's or deontological perspective that causing suffering to any individual to restore a locally or fully extinct species is unethical since it builds on instrumentalization of individuals. In combination with the view that “what has happened to the species has happened,” this line of thought would argue that adding distress on new generations through restoration efforts simply is ethically unjustified, regardless of the species lost. If we decide to act, it can instead be based on a utilitarian weighting where we need to assess the risk of failure, and rate the level and amount of suffering imposed accordingly. Again, we need to address both the potential benefits to biodiversity on a local to global scale, and the potential for suffering by the individual animal.

Human responsibility may also extend long after a successful restoration, depending on the kind of wildlife restored and how humans may/can/will utilize them. Ultimately, however, we believe that we do the future of our world a disservice if we accept extinctions as permanent and rule out all restoration efforts. The future of biodiversity and species conservation depends on humans. This is, after all, Anthropocene, the Time of Man (93, 94), which calls for in-depth reflection on all actions with an impact on individual animals and ecosystems. As with all organisms, everything has consequences (e.g., the “butterfly effect”), but the consequences of human activities for other species are greatest.

In restoration and/or rewilding efforts that include introductions, reintroductions, translocations, supplementary release, or even different forms of re-creation of species [e.g., (66)], we suggest the following 10 ethical points to be considered in a plausibility analysis before decisions are made and action is taken. They can thus be seen as forming a guideline for the decision-making process in restoration and rewilding issues. It is important to note though that we claim no comprehensiveness of the points suggested, but think that if applied, an important step is taken to form general “reintroduction ethics,” as it covers not only value clashes or diverse views regarding content but also how to formulate a strong ethical argument.

However, before we present our 10 points, a few words on how an ethical assessment is often made might be helpful to some readers. In general, ethics here refers to normative issues in applied ethics, i.e., the ambition to analyze what values and issues are at stake, and to formulate what is right or wrong in a certain context, based on the most solid argumentation in order to discern what would be a justified action, i.e., what is the most fundamental principle for the basis to decide what is ethically right to do. This justification can be based on either a principle like in utilitarianism (weighting good vs. bad consequences for all involved, choosing the act generating the overall good for as many as possible) or deontology (focusing on the act itself to be justified as a universal maxim ensuring respect for each ethically relevant entity), or a set of virtues as in virtue ethics, to be reflected upon and related to the specific context. In a well-established eclectic approach, four fundamental ethical principles (autonomy, non-maleficence, beneficence, and justice) are compiled, mirroring both utilitarianism and deontology (95). We suggest it useful to consider the following 10 ethical points:

1. *Description of the situation and problem formulation*. Here, a three step-process is useful. (A) An analysis of the situation is essential to create a clear and value-neutral description of the issues at stake, e.g., “Species x is not present in this area, whereas species y and z are,” or “this situation causes much/little pain and suffering to x individuals.” (B) The causative factors underlying the disappearance or levels of suffering then need to be considered. It is more difficult to remain value-neutral in this phase, due to the frequently large number of plausible and interacting causes and theories. If so, the different possible causes should be listed. (C) Finally, the actual problem should be formulated in a precise and concise way, e.g., “Due to low numbers of grazers in area x, grazing-dependent plants are disappearing.”
2. *Alternatives*. What are the alternatives, e.g., maintaining the status quo or some form of action? If action, what measures can be taken? The answer here is related to the listed causes (1) and the purpose (3 below), and will be value- and perspective-dependent, but also limited to factual possibilities. Can we, for example, facilitate spontaneous recolonization from nearby areas? Can different parts of the landscape or terrain be bridged in some way to increase access and spontaneous transgression? Or are there ecological equivalents available? The potential achievements from actions should be weighed against the counterfactuals, as described by, e.g., Corlett (96). Rather than evaluating success against a fixed baseline, the results of interventions to restore wildlife would then be compared with a counterfactual, i.e., what would have occurred without the interventions.
3. *Purpose*. What is the primary purpose behind any intended action? What arguments make this a valuable purpose and how is it weighed in an ethical framework considering alternatives?
4. *Object of concern*. Scrutiny of the intended species, its specific biological and behavioral needs, prerequisites for the action proposed, as well as the underlying reasons for its disappearance (as above). On a more ethical note, reflection on what the animal represents (on a scale from a commodity to an awe-inspiring creature valuable for its own sake), how to view the moral status of species and individual animals respectively, as well as the potential ethical and societal value of the existence, of having the species in the given area.
5. *Animal welfare*. Further, issues of welfare are relevant and one has to consider actual level of pain, frustration, and harm caused to the object(s) in question. Zoological and especially ethological knowledge and skills are called for here in order to ensure individual capacities are considered in its own right. This is important both to map current welfare of the animals in question and to foresee risks and impairments of welfare.
6. *Potential value clashes*. It is relevant to explore to what extent suffering and welfare matters ethically in relation to other values such as biodiversity or a stable population of a species much depending on (a) actual location and eventually housing of the animals, e.g., limiting their free movement (adaptation and survival possibilities); (b) whether upholding the welfare of wild animals should be less demanding than ensuring domesticated animal welfare levels; and, of course, (c) legislation (in some countries, wild animals are included in animal welfare law; in others, they are not).
7. *Chances of success*. An ethical cost (harm)–benefit analysis for achieving the specific goals of the proposed action should be performed. It is important to include both costs and benefits at the same level of detail, and to be open-minded in selection of factors not supporting one's own view.
8. *Unforeseen consequences*. Although action and risk of failure must sometimes be given priority over passivity and permanent loss, where possible and plausible, a consequence analysis should be conducted for specimens, targeted species, affected species, affected ecosystems, and affected people, before action is taken. The difference between classical conservation, which typically involves negative actions (e.g., preventing something from happening), and rewilding, which typically has a positive trajectory [e.g., re-establishing something; (97)], must be considered. The concept of restoration is in itself an anomaly; we cannot in fact restore a species to any ancestral state, but only form novel trajectories for evolution. This is a challenge for any analysis that aims to consider unforeseen consequences.
9. *Choice of method*. Given solid argumentation for a specific purpose and a certain action, careful selection of “best practice” is needed. For example, if hunting is a primary cause of decreased population, should hunting be regulated to uphold sustainable hunting practices? What, then, does “sustainable” mean for the affected species? Or should hunting be banned? If old-growth deadwood is scarce, what change is needed in forestry practice to ensure it will be provided in sufficient amounts over time? Here, again, the object of concern and its needs as well as potential harms (5) and (unforeseen) consequences (8) related to the method should be considered, in order to investigate the balance of intended benefits.
10. *Adaptivity*. As in research, trial and error is the only way forward. The points above (particularly 2, 7, 8, and 9) are crucial to ensure that potential risks are minimized, but we can never foresee all consequences of all actions; mistakes will be made, lucky coincidences may lead forward, methods can be improved, and actions can be made better. Constant evaluation and re-evaluation, even long after a successful project is undertaken, is a necessary part of any restoration or rewilding project. Hence, there will be a need to describe the situation and reformulate purposes throughout the rewilding process, in a continuous process.

We hope that the aspects advocated and suggestions and thoughts provided here offer guidelines for management efforts aimed at restoring wildlife or wilderness, or simply food for thought for future research efforts in this important field. Finally, we again emphasize that action is necessary to halter the loss of global biodiversity. Passivity is not a value-neutral choice. Since nature and conditions are constantly changing, choosing passivity means choosing further losses. As with the climate consequences of human activities, we have a responsibility to future generations of humans to preserve a rich, sustainable, and diverse planet. Rewilding can provide the benefits of food, experiences, and ecosystem function, and may very well be an important first step toward achieving such a planet.

## Author Contributions

C-GT and HR contributed equally to developing and writing the manuscript.

### Conflict of Interest

The authors declare that the research was conducted in the absence of any commercial or financial relationships that could be construed as a potential conflict of interest.
